# Effective and Selective Recovery of Precious Metals by Thiourea Modified Magnetic Nanoparticles

**DOI:** 10.3390/ijms14059834

**Published:** 2013-05-08

**Authors:** Tai-Lin Lin, Hsing-Lung Lien

**Affiliations:** Department of Civil and Environmental Engineering, National University of Kaohsiung, Kaohsiung City 811, Taiwan; E-Mail: tailin.lin1987@gmail.com

**Keywords:** magnetic nanoparticle, thiourea, precious metals, reuse, recycle, green material, adsorption, nanotechnology

## Abstract

Adsorption of precious metals in acidic aqueous solutions using thiourea modified magnetic magnetite nanoparticle (MNP-Tu) was examined. The MNP-Tu was synthesized, characterized and examined as a reusable adsorbent for the recovery of precious metals. The adsorption kinetics were well fitted with pseudo second-order equation while the adsorption isotherms were fitted with both Langmuir and Freundlich equations. The maximum adsorption capacity of precious metals for MNP-Tu determined by Langmuir model was 43.34, 118.46 and 111.58 mg/g for Pt(IV), Au(III) and Pd(II), respectively at pH 2 and 25 °C. MNP-Tu has high adsorption selectivity towards precious metals even in the presence of competing ions (Cu(II)) at high concentrations. In addition, the MNP-Tu can be regenerated using an aqueous solution containing 0.7 M thiourea and 2% HCl where precious metals can be recovered in a concentrated form. It was found that the MNP-Tu undergoing seven consecutive adsorption-desorption cycles still retained the original adsorption capacity of precious metals. A reductive adsorption resulting in the formation of elemental gold and palladium at the surface of MNP-Tu was observed.

## 1. Introduction

Electrical and electronic wastes (e-wastes) have been recognized as a one of the priority issues of environmental protection since a fast pace of electrical and electronic products was created by the rapid advances of e-technology in the modern society [1]. The e-wastes generally contain high value precious metals such as gold, platinum and palladium and consequently recycling and reuse have become one of the promising options for the treatment of the e-wastes [2]. In general, the e-wastes are treated by aqua regia to dissolve the precious metals. The aqua regia leachate requires further separation and recovery for collecting the precious metals. As a result, the technologies that possess the high selectivity and adsorption capacity towards precious metals are needed.

Currently, many kinds of selective adsorbents have been used for the recovery of precious metals [3–10]. Magnetic separation has been applied as an effective method for solid-liquid phase separation techniques in the wastewater treatment and environmental pollution control [10–14]. For example, the application of an external magnetic field to separate magnetic activated sludge has been developed in the wastewater treatment [13]. Magnetic nanoparticles (MNPs) with high surface areas such as nanoscale magnetite (Fe_3_O_4_) have been considered as good support materials [15]. The surface modified MNPs with specific functional groups such as dendrimer and dimethylamine have been tested for removal of heavy metals because of their high adsorption capacity, short adsorption time and the easy separation of metal-loaded MNPs by applying an external magnetic field [11,14]. On the other hand, functional groups containing sulfur and nitrogen donor atoms are highly selective towards precious metals based on the theory of hard and soft acids and bases (HSAB theory) by Pearson [16]. For example, thiourea [17,18], thiourea-formaldehyde [6], melamine-formaldehyde-thiourea [19], aminopropyls [20,21] and thiolpropyl groups [22] were used to functionalize the surface of supports for the effective adsorption of precious metals.

In this study, we developed thiourea modified magnetic magnetite nanoparticles (MNP-Tu) for magnetic separation and recovery of precious metals from acidic aqueous solutions. The magnetic separation makes MNP-Tu readily to be operated and minimizes the production of the secondary waste caused by the nanoparticles themselves. Gold(III), palladium(II) and platinum(IV) were tested because they are extensively used in many ordinary and advanced applications such as jewelry and ornaments, electrical and electronic devices, catalysts and medical instruments, and many of them inevitably become e-wastes. The objective of this work is aimed at investigating the adsorption properties of MNP-Tu including the adsorption kinetics, capacity, selectivity, efficiency and reusability.

## 2. Results and Discussion

### 2.1. Characterization of Thiourea Modified Magnetic Nanoparticles (MNP-Tu)

Characterization of MNP-Tu was conducted by X-ray diffraction (XRD), Fourier transform infrared spectra (FTIR) and Brunauer-Emmett-Teller (BET) surface analysis. The BET analysis indicated that the specific surface area of MNP and MNP-Tu was 81.4 and 64.3 m^2^/g, respectively. The decrease in surface areas can be attributed to the aminosilanization of MNP where aminosilanes were anchored onto the inner pore volume of MNPs. The aminosilanization of other nanoparticles exhibited similar behavior has also been reported [11,23,24]. It should be noted that the aminosilanization is a key step to stabilize thiourea at the surface of MNPs because it forms a layer of aminosilanes to serve as a platform for further linking thiourea onto the nanoparticles.

**Figure 1:**
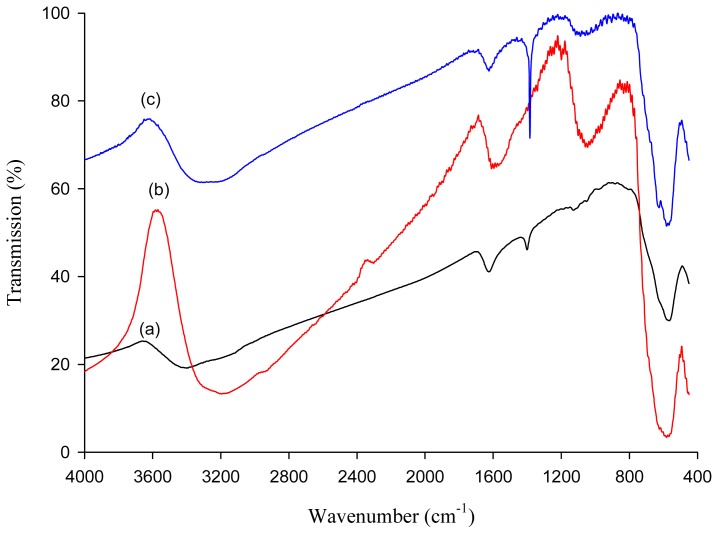
FTIR spectra of (**a**) MNP, (**b**) MNP-Tu and (**c**) Au(III)-loaded MNP-Tu.

The FTIR spectra of MNP, MNP-Tu and metal-loaded MNP-Tu are illustrated in Figure 1. A characteristic adsorption band at 579 cm^−1^ attributed to the Fe-O bond of magnetite was observed in all four samples [11,25]. The absorption band near 1630 cm^−1^ refers to the vibration of remainder H_2_O in the MNP. For MNP-Tu, a strong band appearing at 1620 cm^−1^ is assigned to the NH_2_ bending vibration of the thiourea while another strong band observed at 1082 cm^−1^ is assigned to a motion consisting of symmetric C-N stretching, NH_2_ rocking and C=S stretching [26]. This indicated that thiourea was successfully coated onto the surface of MNP. After adsorption of precious metals, a new band found at 1382 cm^−1^ for the metal-loaded MNP-Tu corresponding to the 1414 cm^−1^ band of pure thiourea, is assigned to the NH_2_ rocking vibration, N-C-N and C=S stretching vibrations [26]. This band indicated that thiourea-metal complexes were formed at the MNP-Tu surface.

**Figure 2:**
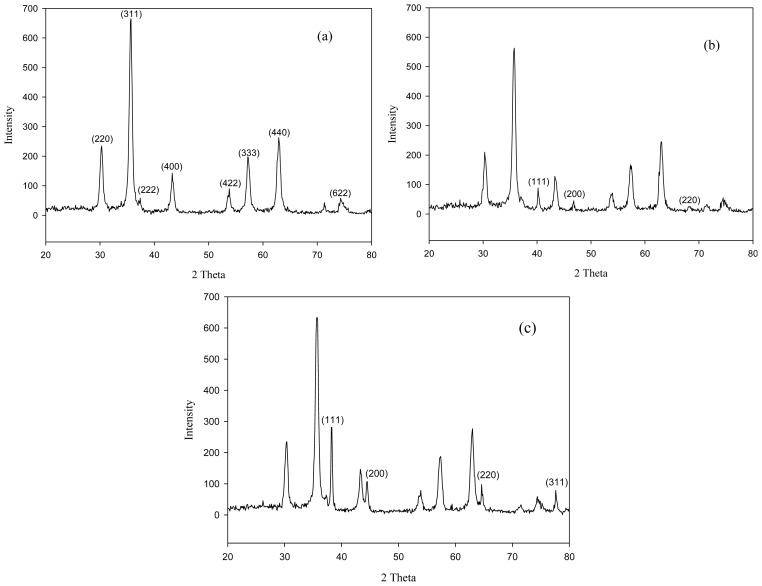
XRD patterns of (**a**) MNP-Tu, (**b**) Pd(II)-loaded MNP-Tu and (**c**) Au(III)-loaded MNP-Tu.

Figure 2 shows the results of XRD analysis for MNP-Tu and metal-loaded MNP-Tu. The XRD pattern of MNP-Tu exhibited diffraction peaks of (220), (311), (222), (400), (422), (333), (440) and (622), which are consisted with The Joint Committee on Powder Diffraction Standards (JCPDS) reference pattern of magnetite (No. 89-4319) with a cubic spinel structure. After the adsorption of Pd(II) and Au(III), the metal-loaded MNP-Tu showed a different pattern of the XRD analysis. In the case of Pd(II)-loaded MNP-Tu, the same peaks assigned to the magnetite was still observed in the XRD pattern; nevertheless, three new peaks (1 1 1) (2θ = 40.1), (200) (2θ = 46.7) and (220) (2θ = 68.1) appearing in the XRD pattern were assigned to the elemental palladium (Pd(0)) (Figure 2b). Analogously, the XRD pattern of Au(III)-loaded MNP-Tu exhibited similar results. The peaks appearing at (1 1 1) (2θ = 38.2), (200) (2θ = 44.4), (220) (2θ = 64.5) and (311) (2θ = 77.6) were assigned to the elemental gold (Au(0)) (Figure 2c). The observation of elemental palladium and gold after the adsorption by MNP-Tu indicated MNP-Tu possesses the reducing capacity for the reduction of precious metallic ions.

Similar results have been documented for the reduction of Au(III) to Au(0) using adsorbents modified with different functional groups including dimethylamine [4], primary amine, ethylenediamine [7], 3-amino-1,2-propanediol [5] and thiolpropyls [22]. The XRD analysis indicated the elemental gold was formed at the surface of persimmon waste modified with dimethylamine in the adsorption of Au(III) [4] while the aggregates of gold was directly observed in a glass reactor during the adsorption of Au(III) by aminated lignin derivatives [7]. Tetrachloroaurate(III) (AuCl_4_^−^) is a strong oxidizing agent that is readily reduced to Au(I) by thiourea, which serves as the reducing agent [27].

(1) $${\text{AuCl}}_{4}^{-}+4\text{CS}{\left({\text{NH}}_{2}\right)}_{2}+{\text{H}}_{2}\text{O}\to \text{Au}{\left(\text{CS}{\left({\text{NH}}_{2}\right)}_{2}\right)}_{2}^{+}+\text{CO}{\left({\text{NH}}_{2}\right)}_{2}+\text{S}+2{\text{Cl}}^{-}+2\text{HCl}$$

Au(I) may be further reduced to Au(0) under reducing conditions.

(2) $$\text{Au}{\left(\text{CS}{\left({\text{NH}}_{2}\right)}_{2}\right)}_{2}^{+}+{\text{e}}^{-}\to {\text{Au}}^{0}+2\text{CS}{\left({\text{NH}}_{2}\right)}_{2}$$

Xiong *et al.* [4] reported the reductive adsorption of Au(III) to Au(0) at the dimethylamine-modified surface occurred at the oxidation and reduction potential (ORP) of about 200 mV. It should be pointed out that the reduction of Pd(II) to Pd(0) was also found in our study suggesting a stronger reducing condition should be developed. Magnetite nanoparticles were found capable of establishing the reducing conditions at ORP below 0 mV [28].

### 2.2. Adsorption Kinetics

**Figure 3:**
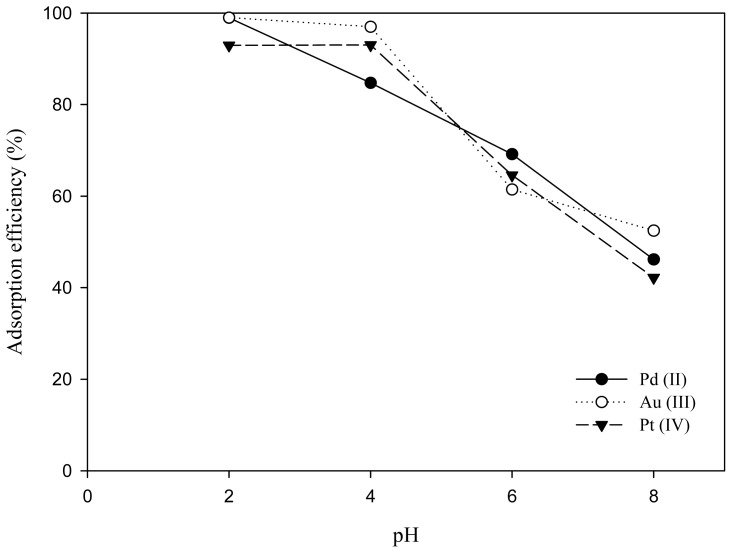
Effects of pH on the adsorption of Pt(IV), Au(III) and Pd (II) by MNP-Tu.

All the experiments were conducted under acidic conditions because the study of pH effects indicated pH is a key factor affecting the adsorption ability. As shown in Figure 3, the highest adsorption efficiency of Pt(IV), Au(III) and Pd(II) by MNP-Tu was about 94%, 99% and 98%, respectively at pH 2 under equilibrium conditions. The optimal adsorption of precious metals with MNP-Tu occurred at pH 2, which is consistent with the previous study that the higher adsorption capacity was obtained at pH 1.0–4.0 [4,17]. It should be noted that the adsorption efficiency of Au(III) and Pd(II) by unmodified MNP was about 30 and 25%, respectively, indicating MNP modified with thiourea enhanced the adsorption affinity of precious metals.

The adsorption kinetics of precious metals on MNP-Tu was analyzed by both pseudo first- and second-order models [29].

(3) $$\frac{{dq}_{t}}{dt}=k_{1}(q_{e}-q_{t})$$

(4) $$\frac{{dq}_{t}}{dt}=k_{2}{\left(q_{e}-q_{t}\right)}^{2}$$

Integrating Equations (3) and (4) for the boundary conditions *t* = 0 to *t* = t and *q_t_* = 0 to *q_t_* = q*_t_*, the equations can be rearranged to a linear form:

(5) $$\text{ln}(\frac{q_{e}-q_{t}}{q_{e}})=-k_{1}t$$

(6) $$\frac{t}{q_{t}}=\frac{1}{k_{2}q_{e}^{2}}+\frac{t}{q_{e}}$$

where *q_t_* and *q_e_* (mg/g) are the amount of metal adsorbed on the MNP-Tu at any time and at equilibrium, respectively; *k*_1_ and *k*_2_ are the first-order and second-order reaction rate constant, respectively; *t* is time (min).

**Figure 4:**
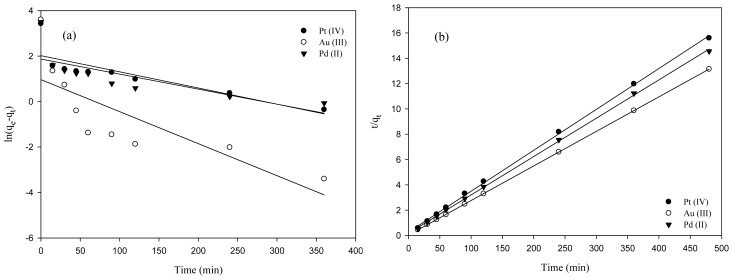
(**a**) Pseudo first-order plots and (**b**) pseudo second-order plots for the adsorption of Pt(IV), Au(III) and Pd(II) on the MNP-Tu at 25 °C.

Figure 4 shows the regression results of adsorption kinetics using pseudo first- and second-order models for the adsorption of precious metals with MNP-Tu. Three different metal ions were tested individually under identical conditions. As shown in Figure 4, the pseudo second-order model fitted the kinetic data better than the first-order model. The coefficient of determination (*R*^2^) for the pseudo first-order model was 0.69, 0.63 and 0.58 in the adsorption of Pt(IV), Au(III) and Pd(II), respectively while it was all greater than 0.99 for the second-order model in the adsorption of each single metal ion with MNP-Tu. This is consistent with the adsorption of precious metals by many other adsorbents such as glycine modified chtiosan resin [8], thiourea modified chtiosan microspheres [17] and 2-(3-(2-aminoethylthio)propylthio)ethanamine modified MNPs [30]. According to the study conducted by Ho and McKay (1999) [29], the observation of second-order kinetics is likely because the rate-limiting step of the adsorption process is chemisorption involving valency forces through sharing or exchange of electrons between metal ions and MNP-Tu.

### 2.3. Adsorption Isotherms

The adsorption capacity of MNP-Tu for various precious metals was determined by adsorption isotherm experiments at 25 °C. Langmuir and Freundlich models are commonly used isotherms that are selected in the study:

(7) $$q_{e}=q_{\text{max}}\frac{K_{L}C_{e}}{1+K_{L}C_{e}}$$

(8) $$q_{e}=K_{f}C_{e}^{1/n}$$

where *q_e_* is the amount of ions adsorbed at equilibrium in mg/g, *C_e_* is the solute equilibrium concentration in mg/L, *q*_max_ and *K_L_* are Langmuir constants indicating the saturated capacity of adsorbents and an energy term, respectively and *K_f_* and 1/*n* are the Freundlich constants.

**Figure 5:**
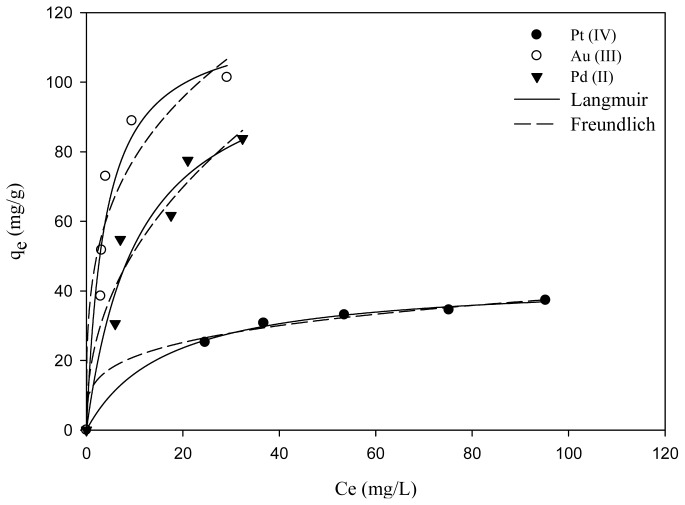
The adsorption isotherms of MNP-Tu for Pt(IV), Au(III) and Pd(II).

Figure 5 shows the experimental data of the adsorption isotherm for Pt(IV), Au(III) and Pd(II) fitted with both the Langmuir and Freundlich equations. Except the fitting of Freundlich model with Au(III), the *R*^2^ of both Langmuir and Freundlich models are all greater than 0.94. Table 1 summarizes the adsorption parameters and the corresponding coefficient of determination. The best-fit experimental data in Langmuir isotherm suggested a monolayer coverage and chemisorption of metal ions at the surface of MNP-Tu, which is in a good agreement of the kinetic study. The n values were between 2.2 and 3.9 indicating that the adsorption of these ions onto MNP-Tu was favorable [5]. The maximum adsorption capacity determined by the Langmuir model for Pt(IV), Au(III) and Pd(II) is 43.34, 118.58 and 111.58 mg/g, respectively. It is worth noting that the relatively high adsorption capacity of MNP-Tu for Au(III) and Pd(II) may be attributed to the formation of elemental gold and palladium confirmed by the XRD analysis (Figure 2b,c).

### 2.4. Competitive Adsorption

**Figure 6:**
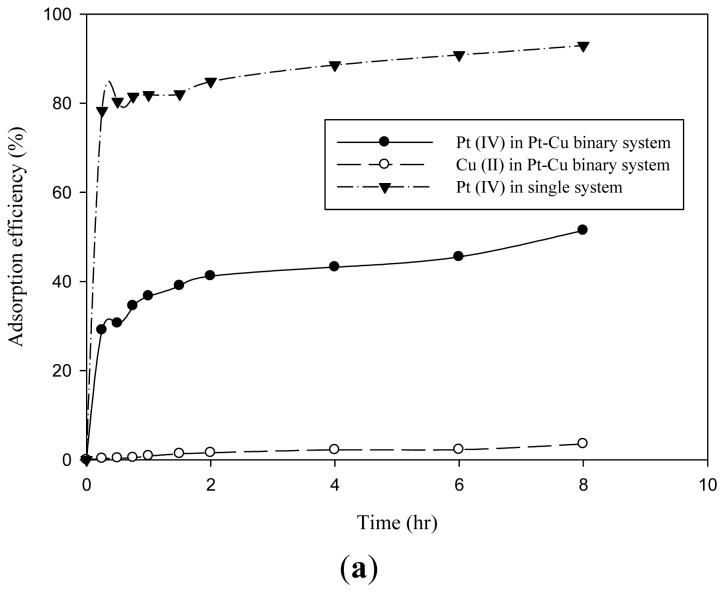

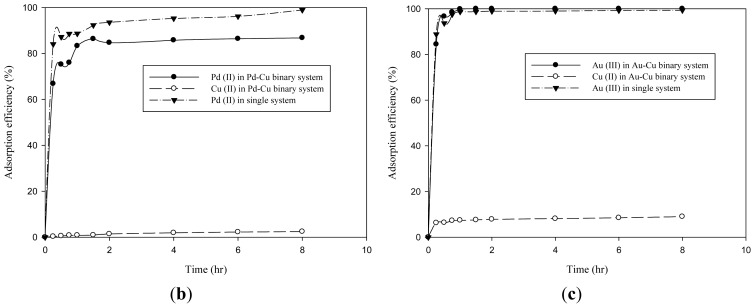
Effects of competing ions on the adsorption of (**a**) Pt(IV), (**b**) Pd(II) and (**c**) Au(III) at MNP-Tu.

Binary competitive adsorption was conducted to investigate the adsorption selectivity of MNP-Tu towards precious metal ions. Copper ions (Cu(II)) were selected to compete with precious metal ions because it is a main component of many electronic wastes containing also precious metals [1,2]. A binary mixture of a precious metal ion and Cu(II) at an equal concentration of 50 mg/L was carried out. The adsorption selectivity was determined according to the study of Sun *et al.* [5].

(9) $${\text{Sel}}_{\text{i}}=\text{log}\frac{{\left({\text{Q}}_{\text{e}}/{\text{C}}_{\text{e}}\right)}_{\text{i}}}{{\left({\text{Q}}_{\text{e}}/{\text{C}}_{\text{e}}\right)}_{\text{j}}}$$

where Q_e_ is the amount of metal adsorbed per unit weight of MNP-Tu at equilibrium (mmol/g); C_e_ is the equilibrium concentration of metal ions in the solution (mM). The index *i* and *j* refer to the precious metal ions and competing ions, respectively. As shown in Figure 6a, the presence of Cu(II) decreased the Pt(IV) adsorption efficiency from 93% to 52%. Cu(II) also exhibited a slight impact on the Pd(II) adsorption efficiency. It can be found that the Pd(II) adsorption efficiency decreased from 99% to 86% in the Cu(II)-Pd(II) binary solution (Figure 6b). Nevertheless, the presence of Cu(II) showed nearly no influence on the Au(III) adsorption efficiency (Figure 6c). Au(III) was readily adsorbed by MNP-Tu in the aqueous solution with or without Cu(II). The adsorption selectivity (Sel_i_) of MNP-Tu towards Pt(IV), Pd(II) and Au(III) was therefore calculated to be 1.46, 2.42 and 5.83, respectively. The high adsorption selectivity of MNP-Tu for Au(III) is attributed to the surface modified thiourea possessing the strong affinity of Au(III). The high selectivity for Au(III) was further confirmed by increasing the concentration of competing ions to 200 mg/L. The adsorption selectivity of MNP-Tu for Au (III) only slightly decreased to 4.81, which was still higher than the use of polystyrene-supported 3-amino-1,2-propanediol as the adsorbent [5]. The competitive adsorption between Au(III) and Cu(II) by various adsorbents has extensively been reported [5,22,31]. The high adsorption selectivity is consistently attributed to the high affinity of Au(III) for the specific functional groups such as amines and thiolpropyls according to the Pearson’s HSAB theory [5,22,31], which is in a good agreement with our study. However, it is worth pointing out that another possibility for the adsorption of the small amount of Cu(II) is the reduction of Cu(II) to Cu(0) occurring at the adsorbent surface [22].

### 2.5. Desorption and Reusability

**Figure 7:**
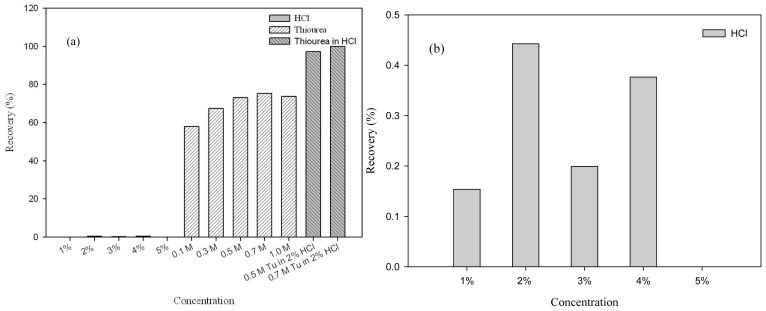
(**a**) Effects of various eluents and concentrations on the desorption of Au(III) from MNP-Tu; (**b**) An enlarged figure from (a) showing the concentration effects of HCl.

The recovery of precious metals from the MNP-Tu adsorption and the reusability of MNP-Tu were conducted by a consecutive adsorption-desorption test. Many eluents including H_2_SO_4_, HCl, HNO_3_, EDTA and thiourea have been reported for the desorption of precious metals [4,7,14,17]. In this study, the desorption of Au(III)-loaded MNP-Tu was tested by using acidic thiourea solutions as the eluent. As shown in Figure 7, the use of HCl alone showed nearly no ability to desorb Au(III). The desorption of Au(III) from the MNP-Tu to the solution was less than 1% when HCl was applied at the concentration of 1%–5%. The Au(III) desorption increased significantly to 50%–75% when thiourea was used. Further, a mixture of thiourea and HCl was found capable of desorbing Au(III) effectively from the surface of MNP-Tu. The Au(III) desorption efficiency reached to nearly 100% when the mixture contained 0.7 M thiourea and 2% HCl.

**Figure 8:**
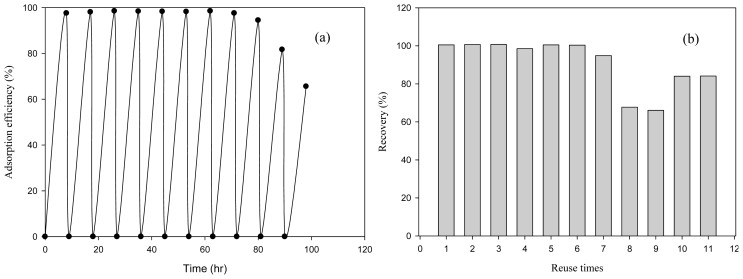
Multi-cycle adsorption-desorption experiments. (**a**) The adsorption efficiency of regenerated MNP-Tu for Au(III) adsorption; (**b**) The recovery efficiency of Au(III) from the Au(III)-loaded regenerated MNP-Tu.

Multicycle adsorption-desorption experiments were conducted by repetitive spiking of Au(III) into a batch bottle to investigate the reusability of MNP-Tu. The regeneration of MNP-Tu was carried out by mixing the Au(III)-loaded MNP-Tu with 10 mL of the acidic thiourea mixture. The results of an experiment during which Au(III) (50 mg/L) was repeatedly spiked into a 250 mL batch bottle containing 1.0 g/L of MNP-Tu are shown in Figure 8. The regenerated MNP-Tu still exhibited high adsorption efficiency to Au(III) after eight cycles of reuse (Figure 8a). The regenerated MNP-Tu retained 99% adsorption efficiency during the first eight cycles and gradually decreased to 70% at the eleventh cycle. Figure 8b shows the recovery of Au(III) from the desorption of Au(III)-loaded MNP-Tu during each cycle of reuse where Au(III) was recovered in a concentrated form. It was observed that Au(III) was readily desorbed in the first seven cycles by using the eluent (0.7 M thiourea and 2% HCl) and the recovery of Au(III) has an average greater than 95% in seven consecutive cycles. The recovery ratio decreased after the eighth cycle, which is similar to the tendency of adsorption efficiency for regenerated MNP-Tu. This suggested that the surface structure of MNP-Tu may be deteriorated after the intensive reuse.

## 3. Experimental Section

### 3.1. Materials

Reagent grade thiourea (99%), hydrogen tetrachloroaurate(III) trihydrate (99%), and potassium hexachloroplatinate(IV) (99%) were purchased from Alfa Aesar. 3-Aminopropyltrimethoxysilane [H_2_N(CH_2_)_3_Si(OCH_3_)_3_, APTS] and glataraldehyde solution (50% in water) were obtained from Fluka. Ferric chloride (FeCl_3_·6H_2_O) and ferrous sulfate (FeSO_4_·7H_2_O) were purchased from SHOWA. Methanol, ethyl alcohol, ammonium hydroxide and palladium(II) chloride were obtained from Aldrich. All chemicals were reagent grade or above and used without further purification.

### 3.2. Preparation of Magnetic Nanoparticles (MNPs)

Nanoscale magnetite (Fe_3_O_4_) was selected as the magnetic nanoparticles in this study. The preparation of nanoscale magnetite was based on the method by which Fe(II) and Fe(III) ions were co-precipitated with ammonia solution and treated under hydrothermal conditions [32]. The detailed procedures can be found in our previous work [11].

### 3.3. Synthesis of Thiourea Modified Magnetic Nanoparticles (MNP-Tu)

**Scheme 1:**
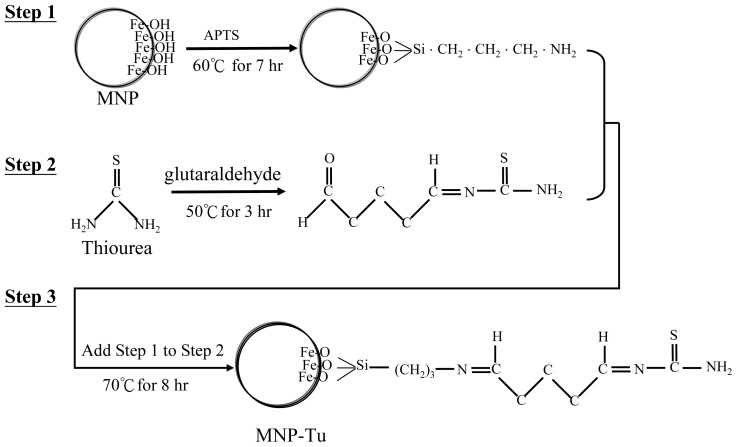
Proposed procedures for the synthesis of MNP-Tu.

After synthesis of MNPs, a 200 mL ethanol mixture containing 2 g MNPs was placed in a round-bottom flask with a condenser. The synthesis and immobilization steps are illustrated by Scheme 1. 10 mL APTS (H_2_N(CH_2_)_3_Si(OCH_3_)_3_) was added and stirred at 60 °C for 7 h to form the APTS modified MNPs. The colloids were then washed by methanol and dried at room temperature by N_2_. Thiourea (4.7 g) was dissolved in 94mL deionized water in a round flask and then 26.7 mL of glutaraldehyde solution (50%) was added to the thiourea solution. The mixture was heated on a water bath for 3 h at 50 °C. After completion of the reaction, then mixture was added with a 50 mL aqueous solution containing 2 g of APTS modified MNPs in the flask and heated for 8 h at 70 °C. The MNP-Tu colloids were washed several times with 0.1 M sodium hydroxide, deionized water and acetone, respectively.

### 3.4. Batch Tests

All equilibrium adsorption experiments were carried out individually for Au(III), Pt(IV) and Pd(II) in a 250 mL high density poly(ethylene), HDPE, vessel containing 0.15 g MNP-Tu in 100 mL aqueous solution at 25 ± 1 °C. The vessel was placed on a rotary shaker at 170 rpm. The solution was adjusted to pH 2 at the beginning of the reaction and monitored periodically throughout the experiment. The solution pH was adjusted by 1 M HCl or NaOH. The initial concentration of precious metals was 50 mg/L for the study of adsorption kinetics and was in a range of 50–110 mg/L for the adsorption isotherm studies. Repetitive adsorption-desorption studies of Au(III) were carried out to evaluate the reusability of MNP-Tu. For each cycle, 50 mg/L of Au(III) solution was added in 100 mL aqueous solutions in the presence of 0.15 g MNP-Tu. After the adsorption reached equilibrium, MNP-Tu was separated by an external magnetic field and the desorption was conducted by mixing Au(III)-loaded MNP-Tu with 10 mL of the eluent for 60 min. Various eluents were tested in the desorption process including HCl, thiourea and the mixture of HCl and thiourea under at different concentrations. After the Au(III) desorption was completed, the regenerated MNP-Tu was magnetically collected and washed thoroughly with deionized water for adsorption in the succeeding cycle. The concentration of Au(III) that was concentrated in the eluent was then analyzed.

### 3.5. Metallic Ion Analysis

Analysis of precious metal ions was conducted by using an inductively coupled plasma-optical emission spectrometry (ICP-OES, PerkinElmer Optima 2000DV, PerkinElmer Inc., Shelton, CT, USA). Prior to analysis, samples were filtered through 0.2 μm filters and acidified with 0.48 M HNO_3_. The ion concentration determined by ICP is the result of triplicate analysis with relative standard deviation less than 1%. Analyses of duplicate samples indicated a relatively analytical error of less than 5% for metal concentrations.

### 3.6. Characterization

The surface characterization of MNP-Tu was conducted by using XRD, FTIR and BET surface analysis. XRD measurements were performed on a X-ray diffractometer (Siemens D5000) using Cu K_α_ radiation producing X-ray with a wavelength of 1.54056 Å. Samples were scanned from 20° to 80° (2θ) at a rate of 2° 2θ/min. FTIR analysis was recorded on a Spectrum GX FTIR spectrometer (Perkin Elmer Inc., Shelton, CT, USA). Specific surface areas of MNP-Tu were determined by BET-N_2_ method using a COULTER SA 3100 surface area analyzer (Coulter Co., Miami, FL, USA). Analysis of the specific surface area was conducted in triplicate.

## 4. Conclusions

In this work, we present a novel magnetic nanoadsorbent for effective and selective adsorption of gold(III), palladium(II) and platinum(IV) in acid aqueous solution. The magnetic nanoadsorbent was prepared by coating thiourea onto the surface of magnetite nanoparticles (MNP-Tu). MNP-Tu effectively adsorbed precious metals at a short contact time (less than 30 min). The maximum adsorption capacity of precious metals for MNP-Tu determined by Langmuir model was 43.34, 118.46 and 111.58 mg/g for Pt(IV), Au(III) and Pd(II), respectively at pH 2 and 25 °C. The adsorption selectivity of MNP-Tu for Au(III) was very high even in the presence of high concentrations of competing Cu(II). Regeneration of MNP-Tu can be achieved by using a mixture of 0.7 M thiourea and 2% HCl as the eluent. A multi-cycle adsorption-desorption test indicates that MNP-Tu is reusable for the recovery of precious metals.

## Figures and Tables

**Table 1:** Langmuir and Freundlich constants of MNP-Tu adsorption for various precious metals.

	Langmuir model			Freundlich model		
		
	*q*_max_ (mg/g)	*K_L_* (L/mg)	*R*^2^	*K_f_*	*n*	*R*^2^
Pt(IV)	43.34	0.06	0.9975	11.73	3.92	0.995
Au(III)	118.46	0.26	0.946	40.13	3.45	0.9076
Pd(II)	111.58	0.09	0.9457	18.83	2.29	0.9422

## References

[1] Widmer R., Oswald-Krapf H., Sinha-Khetriwal D., Schnellmann M., Böni H. (2005). Global perspectives on e-waste. Environ. Impact Asses. Rev.

[2] Cui J., Zhang L. (2008). Metallurgical recovery of metals from electronic waste: A review. J. Hazard. Mater.

[3] Chen X., Lam K.F., Mak S.F., Yeung K.L. (2011). Precious metal recovery by selective adsorption using biosorbents. J. Hazard. Mater.

[4] Xiong Y., Adhikari C.R., Kawakita H., Ohto K., Inoue K., Harada H. (2009). Selective recovery of precious metals by persimmon waste chemically modified with dimethylamine. Bioresour. Technol.

[5] Sun C., Zhang G., Wang C., Qu Q., Zhang Y., Gu Q. (2011). A resin with high adsorption selectivity for Au(III): Preparation, characterization and adsorption properties. Chem. Eng. J.

[6] Ertan E., Gulfen M. (2009). Separation of gold(III) ions from copper(II) and zinc(II) ions using thiourea-formaldehyde or urea-formaldehyde chelating resins. J. Appl. Polym. Sci.

[7] Parajuli D., Kawakita H., Inoue K., Funaoka M. (2006). Recovery of gold(III), palladium(II), and platinum(IV) by aminated lignin derivatives. Ind. Eng. Chem. Res.

[8] Ramesh A., Hasegawa H., Sugimoto W., Maki T., Ueda K. (2008). Adsorption of gold(III), platinum(IV) and palladium(II) onto glycine modified crosslinked chitosan resin. Bioresour. Technol.

[9] Fujiwara K., Ramesh A., Maki T., Hasegawa H., Ueda K. (2007). Adsorption of platinum (IV), palladium (II) and gold (III) from aqueous solutions onto l-lysine modified crosslinked chitosan resin. J. Hazard. Mater.

[10] Uheida A., Iglesias M., Fontas C., Hidalgo M., Salvado V., Zhang Y., Muhammed M. (2006). Sorption of palladium(II), rhodium(III), and platinum(IV) on Fe_3_O_4_ nanoparticles. J. Colloid Interf. Sci.

[11] Chou C.-M., Lien H.-L. (2011). Dendrimer-conjugated magnetic nanoparticles for removal of zinc(II) from aqueous solutions. J. Nanopart. Res.

[12] Lo I.M.C., Hu J., Chen G., Zhang T.C., Surampalli R.Y., Lai K.C.K., Hu Z., Tyagi R.D., Lo I.M.C. (2009). Iron-Based Magnetic Nanoparticles for Removal of Heavy Metals from Electroplating and Metal-Finishing Wastewater. Nanotechnologies for Water Environment Applications.

[13] Hattori S., Watanabe M., Sasaki K., Yasuharu H. (2002). Magnetization of activated sludge by an external magnetic field. Biotechnol. Lett.

[14] Zhou L., Xu J., Liang X., Liu Z. (2010). Adsorption of platinum(IV) and palladium(II) from aqueous solution by magnetic cross-linking chitosan nanoparticles modified with ethylenediamine. J. Hazard. Mater.

[15] Ngomsik A., Bee A., Draye M., Cote G., Cabuil V. (2005). Magnetic nano- and microparticles for metal removal and environmental aapplications: A review. C. R. Chim.

[16] Pearson R.G. (1968). Hard and soft acids, HSAB. PartI. Fundamental principles. J. Chem. Educ.

[17] Zhou L., Liu J., Liu Z. (2009). Adsorption of platinum(IV) and palladium(II) from aaqueous solution by thiourea-modified chitosan microspheres. J. Hazard. Mater.

[18] Wang L., Xing R., Liu S., Yu H., Qin Y., Li K., Feng J., Li R., Li P. (2010). Recovery of silver (I) using a thiourea-modified chitosan resin. J. Hazard. Mater.

[19] Birinci E., Gülfen M., Aydın A.O. (2009). Separation and recovery of palladium(II) from base metal ions by melamine-formaldehyde-thiourea (MFT) chelating resin. Hydrometallurgy.

[20] Lam K.F., Fong C.M., Yeung K.L. (2007). Separation of precious metals using selective mesoporous adsorbents. Gold Bull.

[21] Lam K.F., Yeung K.L., McKay G. (2006). An investigation of gold adsorption from a binary mixture with selective mesoporous silica adsorbents. J. Phys. Chem. B.

[22] Lam K.F., Fong C.M., Yeung K.L., Mckay G. (2008). Selective adsorption of gold from complex mixtures using mesoporous adsorbents. Chem. Eng. J.

[23] Andrzejewska A., Krysztafkiewicz A., Jesionowski T. (2004). Adsorption of organic ddyes on the aminosilane modified TiO_2_ surface. Dye. Pigment.

[24] Ye L., Pelton R., Brook M.A. (2007). Biotinylation of TiO_2_ nanoparticles and their conjugation with sstreptavidin. Langmuir.

[25] Pan B.F., Gao F., Gu H.C. (2005). Dendrimer modified magnetic nanoparticles for protein immobilization. J. Colloid Interface Sci.

[26] El-Bahy G.M.S., El-Sayed B.A., Shabana A.A. (2003). Vibrational and electronic studies on some metal thiourea complexes. Vib. Spectrosc.

[27] Groenewald T. (1977). Potential application of thiourea in the processing of gold. J. S. Afr. Inst. Min. Metall.

[28] Crane R.A., Dickinson M., Popescu I.C., Scott T.B. (2011). Magnetite and zero-valent iron nanoparticles for the remediation of uranium contaminated environmental water. Water Res.

[29] Ho Y.S., McKay G. (1999). Pseudo-second order model for sorption processes. Process Biochem.

[30] Jainae K., Sanuwong K., Nuangjamnong J., Sukpirom N., Unob F. (2010). Extraction and recovery of precious metal ions in wastewater by polystyrene-coated magnetic particles functionalized with 2-(3-(2-aminoethylthio)propylthio)ethanamine. Chem. Eng. J.

[31] Qu R., Sun C., Wang M., Ji C., Xu Q., Zhang Y., Wang C., Chen H., Yin P. (2009). Adsorption of Au(III) from aqueous solution using cotton fiber/chitosan composite adsorbents. Hydrometallurgy.

[32] Mehta R.V., Upadhyay R.V., Charles S.W., Ramchand C.N. (1997). Direct binding of protein to magnetic particles. Biotechnol. Tech.

